# Sociopolitical indicators of food insecurity: a study of 25 US states

**DOI:** 10.1017/S1368980026102353

**Published:** 2026-03-26

**Authors:** Rishika Chakraborty, Katelyn F. Romm, Cassidy LoParco, Y. Tony Yang, Carla J. Berg

**Affiliations:** 1 Center for Health Policy and Media Engagement, School of Nursing, The George Washington University, Washington D.C., USA; 2 TSET Health Promotion Research Center, Stephenson Cancer Center; Department of Pediatrics, College of Medicine, University of Oklahoma Health Sciences, Oklahoma City, OK, USA; 3 Department of Prevention and Community Health, Milken Institute School of Public Health, The George Washington University, Washington D.C., USA; 4 George Washington Cancer Center, The George Washington University, Washington D.C., USA

**Keywords:** Food insecurity, Systems perspective, State-level policy, Inequity, Substance use

## Abstract

**Objective:**

Food insecurity (FI) prevalence has increased globally, including in the USA, and disproportionately affects certain subgroups (e.g. women). Both food-related and non-food-related sociopolitical indicators may impact FI rates; however, these associations are underexplored. This study assessed select state-level sociopolitical indicators among states with higher and lower FI rates compared to the national average.

**Design:**

Cross-sectional

**Setting:**

US

**Participants:**

We identified twenty-five states representing lower (*n* 18) and higher (*n* 7) FI prevalence compared to the 2021–2023 US average (12·2 %) and used national data sources to characterise sixteen sociopolitical indicators (selected via prior review) across three categories: (1) proximal to FI (related to food access/income/resources), (2) inequality (contributing to disparities) and (3) tobacco/alcohol/cannabis regulation (may exacerbate/perpetuate financial constraints). We described each indicator and explored their associations (using *t* tests or Fisher’s tests) with state FI status (high *v*. low).

**Results:**

For proximal indicators, low-FI (*v*. high-FI) states had greater food environment scores, nutrition assistance programme participation, minimum wage and insured individuals. For inequality indicators, low-FI (*v*. high-FI) states had narrower gender wage gaps, greater racial equity and more protective policies for sexual/gender minority populations and abortion rights. For substance-related indicators, low-FI (*v*. high-FI) states had higher cigarette taxes and were more likely to have comprehensive smoke-free laws, legalised non-medical cannabis and provisions for expunging/pardoning prior cannabis-related convictions.

**Conclusion:**

Low-FI states had more sociopolitical indicators aimed at improving food access, financial resources, equality and substance use-related regulations. Findings highlight the importance of adopting a holistic, sustainable, multilevel approach to effectively address the broader determinants of FI.

Food insecurity (FI) is a major global health problem, associated with several adverse outcomes including poor physical^(1)^, mental^(2)^ and cognitive health^(3)^, as well as higher healthcare expenditures^(4)^, and affecting roughly 28 % of people worldwide between 2021 and 2024^(5)^. In the USA, the average prevalence of FI across states and the District of Columbia (DC) was 12·2 % in 2021–2023^(6)^. However, state FI rates vary, from 7·4 % in New Hampshire to 18·9 % in Arkansas^(6)^, and have been relatively stable; for example, both before and after COVID-19, some states like Arkansas, Texas and Mississippi had the highest FI rates, while others like New Hampshire, Massachusetts and North Dakota had the lowest^(7)^. Notably, risk factors for state FI rates are complex and may include determinants beyond geographical or population size. For instance, California and Texas are two of the largest states in terms of population and geographical size in the USA but widely differ in FI prevalence rates, thus suggesting other, stronger determinants of FI.

Identifying state-level determinants of FI, particularly modifiable sociopolitical factors, is crucial to inform FI mitigation strategies and may have implications for jurisdictions across levels (e.g. nationally and locally) globally. For instance, proximal indicators of FI like food access and income^(8)^ may be reflected by food environment^(8)^, eligibility to nutrition assistance programmes such as Supplemental Nutrition Assistance Program (SNAP)^(9)^, minimum wage^(10,11)^ and insurance coverage (which reduce out-of-pocket healthcare costs)^(12)^. Other FI determinants may be indirect, such as inequalities contributing to health disparities^(13)^ like the gender wage gap^(14)^, restricted reproductive healthcare access^(15)^ and non-discrimination policies for minoritised racial/ethnic and sexual/gender groups, as these factors can affect income, educational, employment and housing opportunities/access^(13,16)^. Notably, FI disproportionately affects women, minoritised racial/ethnic and sexual/gender groups, households with children, and those who are unemployed, received less education and experience poverty^(6,8)^. Moreover, these subpopulations may be intersecting, for instance, low-income and minoritised racial/ethnic groups are more likely to reside in neighbourhoods with limited grocery store access^(8)^, which is partially rooted in structural racism^(17)^.

Additionally, substance use may exacerbate FI (e.g. by straining finances)^(18)^. Thus, FI rates may be impacted by state-level policies and regulations related to tobacco^(19)^, alcohol^(20)^ and cannabis^(21)^. Notably, non-medical cannabis legalisation may increase access to cannabis^(21)^ but may also address historical disproportionate incarceration of Black individuals^(22)^, who consequently experience greater barriers to employment, housing and nutrition assistance programmes^(23)^.

Existing research has primarily investigated factors proximally related to FI, while broader social and policy conditions that may shape state-level FI are understudied^(24,25)^. Thus, building on previous literature^(8–10,12,26,27)^ and frameworks^(24,25)^, this cross-sectional study explored select state-level sociopolitical indicators (both proximal FI determinants and factors potentially indirectly influencing FI via financial stability, equity and substance use) among states with significantly higher or lower FI prevalence than the US average^(6)^. Findings may shed light on sociopolitical factors that differentiate states with low *v*. high FI prevalence and inform multilevel FI mitigation strategies in the USA and globally.

## Methods

### Food insecurity

State-level FI prevalence rates were obtained from the US Department of Agriculture (USDA), which combined data from 2021 to 2023 to provide reliable state/DC and national FI estimates^(6)^. The USDA uses standard methodology to identify states with statistically significantly different FI prevalence relative to the US average (12·2 %) at the 90 % confidence level (t > 1·645), as recommended by the US Census Bureau^(28)^. Additional methodological details have been published previously^(28)^.

This study included states with significantly higher and lower FI prevalence relative to the US average^(6)^; we considered DC as a ‘state’ in this study. This sample included twenty-five states (including DC): seven had higher FI prevalence (Arkansas [AR], Kentucky [KY], Louisiana [LA], Mississippi [MS], Oklahoma [OK], South Carolina [SC] and Texas [TX]) and eighteen had lower FI (California [CA], Colorado [CO], Hawai’i [HI], Iowa [IA], Massachusetts [MA], Maryland [MD], Minnesota [MN], North Dakota [ND], New Hampshire [NH], New Jersey [NJ], Pennsylvania [PA], Rhode Island [RI], South Dakota [SD], Virginia [VA], Vermont [VT], Washington [WA], Wisconsin [WI] and DC) than the US average. The remaining twenty-six states with FI prevalence similar to the US average were excluded.

### Variables

One PhD-level author extracted data from robust national public data sources on sixteen state-level sociopolitical indicators across three categories: (1) proximal indicators (Food Environment Index (FEI) scores^(29)^, SNAP participation rates^(30)^, minimum wage amount^(31)^, percentage uninsured^(32)^, Medicaid expansion^(33)^ and child tax credit (CTC) programmes^(34)^); (2) inequality indicators (income inequality^(35)^, gender wage gap^(36)^, protective policies for sexual/gender minoritised groups^(37)^, Racial Equity Index (REI) scores^(38)^ and reproductive rights^(39)^); and (3) tobacco, alcohol and cannabis regulation indicators (100 % smoke-free laws^(40)^, cigarette excise taxes^(41)^, non-medical cannabis legalisation^(42)^, cannabis possession-related expungements or pardons^(43)^ and alcohol outlet density regulation^(44)^). Table 1 provides data source, description and operationalisation of each sociopolitical indicator. Data from 2023 (or before) were extracted to coincide with FI prevalence estimates (2021–2023). For all indicators except percentage uninsured and income inequality, higher scores (for continuous) or presence of policies (for dichotomous) indicated intended positive public health impacts (i.e. aimed at improving food access, financial resources, equality and substance use-related regulations). For percentage uninsured and income inequality, lower scores indicated intended positive public health impacts.

**Table 1.** Sociopolitical indicator source, description and operationalisation

Variables	Data source (year)	Description	Operationalisation	
***Proximal indicators***			***Continuous***	***Categorical***
Food Environment Index (FEI) scores	County Health Rankings & Roadmaps (2019, 2022)^(29)^	Access to healthy foods based on the distance from a grocery store; cost and transportation barriers to purchasing food	0–10 (higher = greater access)	N/A
Supplemental Nutrition Assistance Program (SNAP) participation rates	Center on Budget and Policy Priorities (2020)^(30)^	Percentage of eligible individuals participating in SNAP	49–100 %	N/A
Minimum wage amount	US Department of Labor (2023)^(31)^	$7·25 (Federal minimum wage)	$7·25 and above	N/A
Uninsured	State Health Access Data Assistance Center (2023)^(32)^	Percentage of people that were uninsured (US average is 7·9 %)	2–16·4 %	N/A
Medicaid expansion	Centers for Medicare & Medicaid Services (2023)^(33)^	Expansion, that is, coverage for low-income (up to 138 % Federal Poverty Level) adults *v*. no coverage	N/A	Expansion state (1) *v*. no (0)
State Child Tax Credit (CTC) programme	National Conference of State Legislatures (2023)^(34)^	State-level tax credit for households with eligible children; rules set by state and vary widely	N/A	CTC programme (1) *v*. not (0)
***Inequality indicators***				
Income inequality	State Health Access Data Assistance Center (2023)^(35)^	Measures income disparities based on Gini coefficient (US average is 0·48)	0·42–0·52 (higher = greater inequality)	N/A
Gender wage gap (what women were paid for every dollar paid to men)	National Women’s Law Center (2023)^(36)^	Values indicate the amount women were paid for every dollar paid to men (the US average being $0·83: $1)	$0·71–0·89	N/A
Protective policies for sexual/gender minoritised groups	Movement Advancement Project (2020)^(37)^	Overall policy tally across multiple policies shaping Lesbian, Gay, Bisexual, Transgender and Queer (LGBTQ) people’s lives, experiences and equality	N/A	Medium/high policies (1) *v*. fair/low/negative (0)
Racial Equity Index (REI) scores	National Equity Atlas (2022)^(38)^	Assesses 9 indicators of economic vitality, readiness and connectedness, measuring overall population well-being as well as racial gaps in outcomes	19·5–62·4 (higher = greater equity)	N/A
Reproductive rights	Center for Reproductive Rights (2023)^(39)^	Indicating states with and without abortion protections	N/A	Abortion protections (1) *v*. not (0)
***Tobacco, alcohol and cannabis regulation indicators***				
100 % Smoke-free laws	CDC-State Tobacco Activities Tracking and Evaluation System (2023)^(40)^	Implementation of 100 % smoke-free laws in 3 settings – workplaces, bars and restaurants	N/A	States with smoke-free law coverage in any setting (1) *v*. none (0)
Cigarette excise taxes	Campaign for Tobacco Free Kids (2023)^(41)^	Excise taxes per cigarette pack (US average is $1·93)^(50)^	$0·17–$5·35	N/A
Non-medical cannabis legalisation	National Conference of State Legislatures (2023)^(42)^	Indicating legalisation status of non-medical cannabis in the state	N/A	States with legal non-medical cannabis (1) *v*. not (0)
Cannabis possession-related expungements or pardons	National Conference of State Legislatures (2023)^(43)^	Indicating provisions for cannabis possession-related expungements/pardons in the state	N/A	States allowing cannabis possession-related expungements or pardons (1) *v*. not (0)
Alcohol outlet density regulation	ChangeLab Solutions and CDC (2022)^(44)^	States with any state-level regulation controlling alcohol outlet density and without	N/A	States with any state-level regulation controlling alcohol outlet density (1) *v*. no law (0)

### Data analysis

We described continuous and dichotomous sociopolitical indicators for each state based on FI status (low *v*. high) and then conducted exploratory bivariate analyses (*t* tests or Fisher’s tests, as appropriate) characterising them in relation to state FI status. Significance level was set at *P* < 0·05. Given the exploratory nature of these tests, we did not adjust for confounders; findings should be interpreted as hypothesis-generating.

## Results

Table 2 summarises each state’s FI rates and sociopolitical indicators. FI rates in the eighteen low-FI states ranged from 7·4 % in NH to 11·4 % in CA. Rates in the seven high-FI states ranged from 14·4 % in SC to 18·9 % in AR.

**Table 2.** Food insecurity prevalence and relevant state-level sociopolitical indicators among 25 states (including DC) in the USA representing high (*n* 7) and low (*n* 18) food insecurity prevalence

	Proximal indicators							Inequality indicators				
State	FI (%)	FEI^†^	SNAP rates (%)^†^	Min wage ($)^†^	Un-insured (%)^‡^	Medicaid expansion*	Child tax credit*	Income inequality^‡^	Gender wage gap^†^	LGBTQ anti-discrimination laws^†,§^	Racial Equity Index^†^	Reproductive rights*
States with FI prevalence above US average (12·2 %)												
Arkansas	18·9	4·7	62	11·00	8·9	1	0	0·47	0·82	0	35·3	0
Kentucky	14·5	6·8	65	7·25	5·4	1	0	0·48	0·82	1	47·0	0
Louisiana	16·2	4·8	83	7·25	6·9	1	0	0·50	0·71	0	29·9	0
Mississippi	16·2	4·0	62	7·25	10·3	0	0	0·48	0·78	0	19·0	0
Oklahoma	15·4	5·6	84	7·25	11·4	1	1	0·47	0·78	0	51·9	0
South Carolina	14·4	6·7	69	7·25	9·1	0	0	0·47	0·81	0	46·0	0
Texas	16·9	5·9	69	7·25	16·4	0	0	0·48	0·82	0	49·0	0
States with FI prevalence below US average (12·2 %)												
California	11·4	8·6	66	15·5	6·4	1	1	0·49	0·87	4	43·5	1
Colorado	9·9	8·5	76	13·65	6·7	1	1	0·46	0·84	4	60·3	1
District of Columbia	8·8	8·9	93	17·0	2·7	1	1	0·52	0·86	4	57·4	1
Hawai’i	9·6	7·8	83	12·0	3·2	1	0	0·45	0·88	4	59·8	1
Iowa	9·8	8·8	85	7·25	5·0	1	0	0·45	0·81	2	44·2	0
Maryland	10·4	8·8	85	13·25	6·3	1	1	0·46	0·86	3	56·1	1
Massachusetts	9·3	9·2	97	15·0	2·6	1	1	0·49	0·86	4	56·0	1
Minnesota	9·1	9·0	76	10·59	4·2	1	1	0·45	0·85	4	41·6	1
New Hampshire	7·4	9·5	79	7·25	4·9	1	0	0·44	0·76	3	61	0
New Jersey	9·8	9·2	72	14·13	7·2	1	1	0·48	0·83	3	49·9	1
North Dakota	8·6	8·9	66	7·25	4·5	1	0	0·45	0·76	0	31·1	0
Pennsylvania	10·8	8·5	95	7·25	5·4	1	0	0·48	0·81	2	49·8	0
Rhode Island	9·7	8·8	95	13·0	4·5	1	0	0·47	0·89	4	58·8	1
South Dakota	9·0	7·7	80	10·8	8·3	1	0	0·46	0·81	0	36·2	0
Vermont	9·2	9·1	95	13·18	3·4	1	1	0·45	0·88	4	57·3	1
Virginia	10·0	9·0	77	12·0	6·4	1	0	0·47	0·81	1	62·4	0
Washington	9·5	8·5	94	15·74	6·3	1	0	0·47	0·79	4	54·6	1
Wisconsin	10·7	9·1	92	7·25	4·9	0	0	0·45	0·83	2	55·6	0

	Tobacco, alcohol and cannabis regulation indicators				
State	Smoke-free laws^†^	Cigarette tax ($)^†^	Non-medical cannabis legal*	Cannabis conviction, pardons, etc.*	Alcohol outlet density laws*
States with FI prevalence above US average (12·2 %)					
Arkansas	1	1·15	0	0	1
Kentucky	0	1·10	0	0	1
Louisiana	2	1·08	0	0	0
Mississippi	0	0·68	0	0	0
Oklahoma	0	2·03	0	0	0
South Carolina	0	0·57	0	0	0
Texas	0	1·41	0	0	0
States with FI prevalence below US average (12·2 %)					
California	3	2·87	1	1	1
Colorado	3	1·94	1	1	1
District of Columbia	3	4·50	1	1	1
Hawai’i	3	3·20	0	1	0
Iowa	3	1·36	0	0	0
Maryland	3	3·75	1	1	1
Massachusetts	3	3·51	1	1	1
Minnesota	3	3·04	1	1	1
New Hampshire	1	1·78	0	1	1
New Jersey	3	2·70	1	1	1
North Dakota	3	0·44	0	1	0
Pennsylvania	1	2·60	0	1	1
Rhode Island	3	4·25	1	1	1
South Dakota	3	1·53	0	0	1
Vermont	3	3·08	1	1	0
Virginia	0	0·60	1	1	0
Washington	3	3·03	1	1	1
Wisconsin	3	2·52	0	0	1

FI, food insecurity; FEI, Food Environment Index; SNAP, Supplemental Nutrition Assistance Program; LGBTQ, Lesbian, Gay, Bisexual, Transgender and Queer.

*1 = yes, 0 = no.

^†^Higher = intended positive public health impact.

^‡^Lower = intended positive public health impact.

^§^0 = Negative, 1 = low, 2 = fair, 3 = medium, 4 = high policies.

### Proximal indicators

In low-FI states, FEI scores (range: 0–10, higher = greater access) ranged from 7·7 in SD to 9·5 in NH; SNAP participation ranged from 66 % in CA and ND to 97 % in MA; minimum wage varied from $7·25 (federal minimum) in IA, NH, ND, PA and WI to $17·0 in DC; and percentage uninsured ranged from 2·6 % in MA to 8·3 % in SD. All states but WI (94·4 %, *n* 17/18) had Medicaid expansion programmes. CA, CO, DC, MD, MA, MN, NJ and VT (44·4 %, *n* 8/18) had state-level CTC programmes.

High-FI states varied in FEI scores (from 4·0 in MS to 6·8 in KY), SNAP participation (62 % in AR to 84 % in OK) and percentage uninsured (5·4 % in KY to 16·4 % in TX). Six (85·7 %, *n* 6/7) had the federal minimum wage ($7·25); AR was higher ($11·00). AR, KY, OK and LA (57·1 %, *n* 4/7) had Medicaid expansion. Only OK (14·3 %, *n* 1/7) had state-level CTC programmes.

### Inequality indicators

In low-FI states, Gini coefficients (higher = greater inequality) varied from 0·44 in NH to 0·52 in DC, gender wage gaps ranged from $0·76 in NH and ND to $0·89 in RI and REI scores (range: 19·5–62·4, higher = greater equity) ranged from 31·1 in SD to 62·4 in VA. All but IA, ND, PA, SD, VA and WI (66·7 %, *n* 12/18) had strong protective policies for sexual/gender minority groups, and 61·1 % (*n* 11/18; CA, CO, DC, HI, MA, MD, MN, NJ, RI, VT and WA) had abortion protections.

High-FI states varied in the Gini coefficient (0·47 in AR, OK and SC to 0·50 in LA), gender wage gap (0·71 in LA to 0·82 in AR, KY, TX) and REI scores (19·0 in MS to 51·9 in OK). None had strong protective policies for sexual/gender minority groups or abortion.

### Tobacco, alcohol and cannabis regulation indicators

For low-FI states, cigarette excise taxes varied from $0·44 in ND to $4·50 in DC. All but NH, PA and VA (83·3 %, *n* 15/18) had 100 % smoke-free laws. All but HI, IA, NH, ND, PA, SD and WI (61·1 %, *n* 11/18) had legalised non-medical cannabis; most (83·3 %, *n* 15/18) had provisions for expunging/sealing/pardoning prior cannabis-related convictions. All but HI, IA, ND, VA and VT (72·2 %, *n* 13/18) had alcohol outlet density regulations.

In high-FI states, cigarette excise taxes varied from $0·57 in SC to $2·03 in OK. Only two states (28·6 %, *n* 2/7) had 100 % smoke-free laws (AR and LA) or alcohol outlet density regulations (AR and KY). None had legalised non-medical cannabis nor provisions for expunging/sealing/pardoning prior cannabis-related convictions.

### Exploratory analysis

For proximal indicators, low-FI (*v*. high-FI) states had greater FEI scores, SNAP participation, minimum wage and percentage insured (*P*’s < 0·05, Table 3). For inequality indicators, low-FI (*v*. high-FI) states had narrower gender wage gaps and greater REI scores. A greater proportion of low-FI (*v*. high-FI) states had more protective policies for sexual/gender minority populations and abortion rights (*P*’s < 0·05). For tobacco, alcohol and cannabis regulation indicators, low-FI (*v*. high-FI) states had greater cigarette taxes. A greater proportion of low-FI (*v*. high-FI) states had 100 % smoke-free laws, legalised non-medical cannabis and provisions for expunging/sealing/pardoning prior cannabis-related convictions (*P*’s < 0·05).

**Table 3.** Sociopolitical indicators and characteristics across 25 states (including DC) in the US with low *v*. high food insecurity (FI)

	Total *n* 25		Low FI prevalence *n* 18		High FI prevalence *n* 7		
Sociopolitical indicator	Mean or *n*	sd or %	Mean or *n*	sd or %	Mean or *n*	sd or %	*P*-value
***Proximal indicators***							
Food Environment Index scores	7·86	1·64	8·77	0·46	5·50	1·06	***< 0·001***
Supplemental Nutrition Assistance Program participation rates (%)	80·00	11·49	84·00	10·00	71·00	9·00	***< 0·001***
Minimum wage amount ($)	10·66	3·41	11·78	3·32	7·79	1·42	***< 0·001***
Uninsured (%)	6·45	3·07	5·00	2·00	10·00	4·00	***0·027***
Medicaid expansion enrolment							
Non-expansion state	4	16·00	1	5·56	3	42·86	0·053
Expansion state	21	84·00	17	94·44	4	57·14	
State child tax credit							
No policy	16	64·00	10	55·56	6	85·71	0·355
Has a policy	9	36·00	8	44·44	1	14·29	
***Inequality indicators***							
Income inequality	0·47	0·02	0·47	0·02	0·48	0·01	***0·049***
Gender wage gap *($ women were paid for every $1 paid to men)*	0·82	0·04	0·83	0·04	0·79	0·04	***< 0·001***
Sexual/gender minoritised groups anti-discrimination laws							
Fair/low/negative policies	13	52·00	6	33·33	7	100·00	***< 0·01***
Medium/high policies	12	48·00	12	66·67	0	0·00	
Racial Equity Index scores	48·55	11·25	51·98	9·11	39·73	12·06	***< 0·001***
Reproductive rights							
No abortion protections	14	56·00	7	38·89	7	100·0	***< 0·01***
Abortion protections	11	44·00	11	61·11	0	0·0	
***Tobacco, alcohol and cannabis regulation indicators***							
100 % smoke-free laws							
No coverage	6	24·00	1	5·56	5	71·43	***< 0·01***
Any coverage	19	76·00	17	94·44	2	28·57	
Cigarette excise taxes ($)	2·19	1·19	2·59	1·14	1·15	0·48	***< 0·001***
Non-medical cannabis legalisation							
No	14	56·00	7	38·89	7	100·0	***< 0·01***
Yes	11	44·00	11	61·11	0	0·0	
Cannabis possession-related expungements/pardons							
Not allowed	10	40·00	3	16·67	7	100·0	***< 0·001***
Allowed	15	60·00	15	83·33	0	0·0	
Alcohol outlet density regulation							
No state-level regulation	10	40·00	5	27·78	5	71·43	0·075
Any state-level regulation	15	60·00	13	72·22	2	28·57	

Boldface italics indicates statistical significance (*P* < 0.05).

## Discussion

Relative to high-FI states, low-FI states had more sociopolitical indicators with intended positive public health impacts. Notably, low-FI states had both proximal sociopolitical indicators aimed to improve food access and distal factors influencing inequalities and substance use, which may have had beneficial spillover effects on FI. Thus, findings suggest that FI mitigation efforts should apply a systems perspective^(45,46)^ and consider population-level strategies to increase financial resources, reduce disparities and regulate substance use in addition to strengthening food access. These insights may have implications not only for the USA but globally, as well.

FI mitigation efforts should consider which factors (i.e. proximal, inequality or substance use-related regulations) amplify or reduce FI and the magnitudes of their effects, individually and in the context of other factors. These considerations may explain unexpected findings; for instance, low-FI states like SD or VA have some sociopolitical indicators with low scores for their intended positive public health impacts (such as for SNAP participation rates, wage gap and tobacco regulations). Relatedly, it is important to understand how multiple indicators within the same category may impact FI. For instance, although OK and LA have high SNAP participation rates among eligible participants, they have high FI. It is possible that, despite SNAP participation, poor food environments (which relate to income and healthy food proximity) are more powerful determinants of FI rates in these states. Such differences may also suggest potentially unmeasured or interacting indicators that might influence FI. Thus, future research investigating the magnitude of the impact of these intersecting indicators on FI is warranted.

Notably, findings suggest that non-food-related sociopolitical indicators (that are not traditionally targeted by FI-related policies) may be important in addressing FI, underscoring the increasingly urgent need to prioritise cross-sector policy initiatives. This research reinforces the importance of a Health in All Policies^(47)^ approach, an integrated policymaking framework that considers health implications across all sectors to improve population outcomes^(48)^. Thus, as FI rates continue to rise, sustainable FI mitigation efforts are crucial and must prioritise coordinated action across multiple government agencies and sectors to advance relevant policy initiatives. These may include enhancing healthy food access, expanding SNAP budgets, minimum wages and Medicaid coverage, promoting health equity and strengthening public health protections like substance-related regulations^(47,49)^.

Some aspects of this study warrant careful consideration. Study limitations include an ecological analysis of a small subset (*n* 25) of US states. Thus, findings may not generalise to other states, levels (e.g. individual, local) or international settings. Furthermore, due to the small sample size, we relied on bivariate analyses and did not account for unmeasured heterogeneity across states or potential confounders, for example, other state-level sociopolitical factors (e.g. substance use recovery programme accessibility or SNAP implementation features). Additionally, this study’s cross-sectional, exploratory nature precludes causal inference. Finally, despite use of robust data sources, some sociopolitical indicators may not fully or accurately reflect each state’s complex policy landscapes. Considering these limitations, patterns identified between sociopolitical environment and FI must be interpreted with caution.

## Conclusion

Low-FI states exhibited more sociopolitical indicators with intended positive public health impact, particularly related to food access, financial security, equity and substance use-related regulations. A sustainable, multilevel, holistic approach addressing multiple intersecting determinants of FI, beyond proximal factors alone, is essential. Future research utilising longitudinal designs and examining a broader set multilevel determinants is needed to better understand causal relationships and interactions among sociopolitical indicators of FI.

## Data Availability

All data are publicly available.
